# Bioactive Compounds, Antioxidant, Xanthine Oxidase Inhibitory, Tyrosinase Inhibitory and Anti-Inflammatory Activities of Selected Agro-Industrial By-products

**DOI:** 10.3390/ijms12128610

**Published:** 2011-11-29

**Authors:** Ehsan Oskoueian, Norhani Abdullah, Rudi Hendra, Ehsan Karimi

**Affiliations:** 1 Department of Microbiology, Faculty of Biotechnology and Biomolecular Sciences, University Putra Malaysia (UPM), 43400 UPM Serdang, Selangor, Malaysia; E-Mail: ehs424@yahoo.com; 2 Agriculture Biotechnology Research Institute of Iran (ABRII)-East and North-East Branch, P.O.B. 91735/844, Mashhad, Iran; E-Mails: ehs424@gmail.com (E.O.); ehsan_b_karimi@yahoo.com (E.K.); 3 Department of Biochemistry, Faculty of Biotechnology and Biomolecular Sciences, University Putra Malaysia (UPM), 43400 UPM Serdang, Selangor, Malaysia; E-Mail: rootdee2001@yahoo.com; 4 Institute of Tropical Agriculture, University Putra Malaysia (UPM), 43400 UPM Serdang, Selangor, Malaysia; 5 Department of Chemistry, Faculty of Mathematic and Natural Sciences, University of Riau, 28143 Pekanbaru, Riau, Indonesia; 6 Department of Crop Science, Faculty of Agriculture, University Putra Malaysia (UPM), 43400 UPM Serdang, Selangor, Malaysia

**Keywords:** agro-industrial by-products, bioactive compounds, antioxidant, anti-inflammatory, xanthine oxidase inhibition, tyrosinase inhibition

## Abstract

Evaluation of abundantly available agro-industrial by-products for their bioactive compounds and biological activities is beneficial in particular for the food and pharmaceutical industries. In this study, rapeseed meal, cottonseed meal and soybean meal were investigated for the presence of bioactive compounds and antioxidant, anti-inflammatory, xanthine oxidase and tyrosinase inhibitory activities. Methanolic extracts of rapeseed meal showed significantly (*P* < 0.01) higher phenolics and flavonoids contents; and significantly (*P* < 0.01) higher DPPH and nitric oxide free radical scavenging activities when compared to that of cottonseed meal and soybean meal extracts. Ferric thiocyanate and thiobarbituric acid tests results showed rapeseed meal with the highest antioxidant activity (*P* < 0.01) followed by BHT, cotton seed meal and soybean meal. Rapeseed meal extract in xanthine oxidase and tyrosinase inhibitory assays showed the lowest IC_50_ values followed by cottonseed and soybean meals. Anti-inflammatory assay using IFN-γ/LPS stimulated RAW 264.7 cells indicated rapeseed meal is a potent source of anti-inflammatory agent. Correlation analysis showed that phenolics and flavonoids were highly correlated to both antioxidant and anti-inflammatory activities. Rapeseed meal was found to be promising as a natural source of bioactive compounds with high antioxidant, anti-inflammatory, xanthine oxidase and tyrosinase inhibitory activities in contrast to cotton and soybean meals.

## 1. Introduction

Rapeseed meal, cottonseed meal and soybean meal are common by-products of the oil industry. These by-products are used for biodiesel, fertilizer and feed industries for livestock production, in particular the poultry industry, due to their high protein and low fibre content [1]. Chemical analyses of these oilseed meals showed the presence of lignin, cellulose, amino acids, proteins and polyphenolic compounds [2] which contain functional groups including carboxyl, hydroxyl and methyl groups as well as high amounts of fixed anionic and cationic moieties which may offer therapeutic effects such as antimicrobial, anti-inflammatory, anticancer and antioxidant activities [3–6].

The increasing awareness of consumers to issues regarding food additive safety, results in an enhanced effort in finding alternative additives and preservatives from natural and probably safer sources [7]. In this regard, food manufacturers have been encouraged to use natural antioxidants instead of synthetic compounds to maintain the nutritional values of their products [8]. For instance, commercial antioxidants such as butylated hydroxytoluene (BHT) and butylated hydroxyanisole (BHA) can be replaced by plant extracts particularly polyphenols [7] obtained from agro-industrial by-products. Moreover, in the pharmaceutical industry there are attempts to produce drugs and food supplements based on bioactive compounds obtained from agro-industrial by-products in a sustainable manner [9].

The possible applications of rapeseed, cotton seed and soybean meals as sources of bioactive compounds have not been extensively studied. Therefore, rapeseed meal, cottonseed meal and soybean meal were evaluated for the presence of bioactive compounds and biological activities such as antioxidant, anti-inflammatory, xanthine oxidase and tyrosinase inhibition.

## 2. Results and Discussion

### 2.1. Total Phenolic and Flavonoid Compounds

Phenolics and flavonoids are important groups of biologically active compounds in plants [10]. Table 1 shows the total phenolic and flavonoids content in the methanolic extracts of rapeseed meal, cottonseed meal and soybean meal. The total phenolic content of 5.3 ± 0.02 mg gallic acid equivalents g^−1^ DW and total flavonoid content of 2.3 ± 0.01 mg rutin equivalents g^−1^ DW of rapeseed meal were significantly (*P* < 0.01) higher than that of the cottonseed meal and soybean meal (Table 1). These results were in agreement with Vuorela *et al.* [11] who reported the higher content of phenolic compounds in rapeseed meal when compared to other oilseed meals.

### 2.2. Bioactive Compounds

Due to the diversity and complexity of phenolic and flavonoid compounds in plants, it is rather difficult to characterize every compound and elucidate its structure, but it is not difficult to identify major group of phenolic and flavonoid compounds. The major phenolics and flavonoids components identified based on the standards using high performance liquid chromatography (HPLC) are shown in Table 2. Rapeseed meal contained phenolics including gallic acid (419.5 ± 3.32 μg g^−1^) and syringic acid (177.6 ± 2.82 μg g^−1^) while the flavonoids detected were apigenin (64.9 ± 2.48 μg g^−1^), kaempferol (494.0 ± 1.71 μg g^−1^) and naringenin (793.5 ± 5.90 μg g^−1^). Cotton seed meal did not contain phenolics based on the standards used in this study but flavonoids such as kaempferol (113.7 ± 4.59 μg g^−1^), naringenin (178.9 ± 6.88 μg g^−1^) and rutin (209.1 ± 2.45 μg g^−1^) were observed. The phenolic found in soybean meal was caffeic acid (295.8 ± 2.73 μg g^−1^) while the flavonoids detected were naringenin (352.3 ± 3.66 μg g^−1^) and isoflavonoid was daidzein (521.4 ± 6.58 μg g^−1^). The HPLC results showed that rapeseed meal contained more phenolics and flavonoids compounds as compared to the cottonseed and soybean meals. Xiao *et al.* [12] recently have discussed comprehensively the role of phenolics and flavonoids in relation to their health benefits as anti-inflammatory, antioxidant and anticancer agents, as well as on other effects including free radical scavenging activity, anti-hypertensive effects, coronary heart disease prevention and anti-human immunodeficiency virus infection.

### 2.3. Antioxidant Activity (DPPH and NO Scavenging)

**Figure 1:**
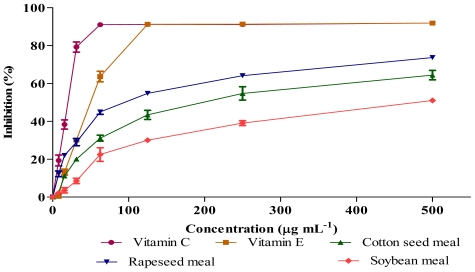
DPPH scavenging activity of meal extracts and vitamins at different concentrations. Each value represents mean ± SEM of three replicates.

**Figure 2:**
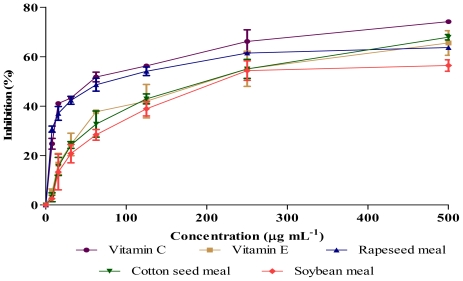
Nitric oxide scavenging activity of meal extracts and vitamins at different concentrations. Each value represents mean ± SEM of three replicates.

Antioxidants are responsible in preventing oxidative damages to the cellular components as a consequence of biochemical reactions. Some phenolics and flavonoids appeared to be more active than vitamins for this purpose and their activities depend on the structure and total number of hydroxyl groups [13]. Figures 1 and 2 show the antioxidant activities of extracts obtained from rapeseed meal, cottonseed meal and soybean meal in the reactions with 1,1-diphenyl-2-picrylhydrazil (DPPH) and nitric oxide (NO), respectively. The extracts inhibited the DPPH and NO in a dose dependent manner. The IC_50_ concentrations (Table 3) showed significant (*P* < 0.01) differences in DPPH and NO scavenging activity among samples, where rapeseed meal showed the lowest value followed by cottonseed meal and soybean meal. Although a crude extract was used in the assay, the IC_50_ value of rapeseed meal extract was about twice of vitamin E (positive control), indicating the presence of highly bioactive compounds in the rapeseed meal extract.

### 2.4. Total Antioxidant Activity (FTC and TBA Tests)

**Figure 3:**
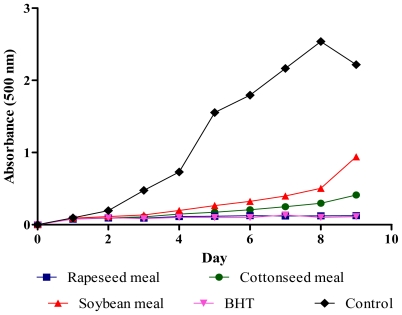
Hydroperoxides inhibitory activity of meal extracts and butylated hydroxytoluene (BHT) by ferric thiocyanate test.

**Figure 4:**
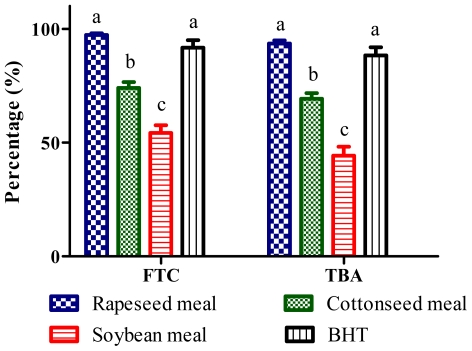
Percentage inhibition of linoleic acid peroxidation measured by the ferric thiocyanate test (FTC) and thiobarbituric acid (TBA) antioxidant assays.

Hydroperoxides inhibitory activity of meal extracts in ferric thiocyanate test (FTC) test is presented in Figure 3. Almost all extracts significantly (*P* < 0.01) reduced the hydroperoxides formation in the linoleic acid emulsion throughout the incubation period when compared to the negative control. The percentage inhibitions of the extracts in the last day of the assay were 97.2%, 74.1%, 54.2% and 91.7% for rapeseed, cottonseed, soybean and butylated hydroxytoluene (BHT), respectively (Figure 4). The rapeseed meal extract showed similar percentage inhibition value to that of BHT at the end of the incubation period, indicating rapeseed meal extract possesses antioxidative potential equivalent to that of BHT. Thiobarbituric acid (TBA) test determined the content of thiobarbituric acid reactive substances at the end of lipid oxidation. Rapeseed, cottonseed, soybean meals extracts and BHT inhibited thiobarbituric acid reactive substances by 93.5%, 69.2%, 44.1% and 88.3%, respectively (Figure 4). The results showed that rapeseed meal exhibited the strongest activity (*P* < 0.01) as compared to the other extracts. The result suggests that rapeseed contains antioxidative compounds as shown in Table 2 which react aggressively toward hydroxyl radicals and retard the formation of hydroperoxides.

### 2.5. Xanthine Oxidase (XO) Inhibitory Activity

**Figure 5:**
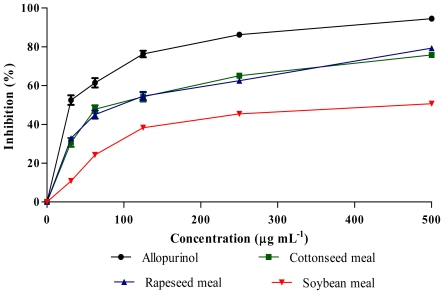
Xanthine oxidase inhibitory activity of meal extracts and allopurinol at different concentrations. Each value represents mean ± SEM of three replicates.

The extracts XO inhibitory activities are presented in Figure 5. Inhibition of XO led to a decrease in production of uric acid, which was determined spectrophotometrically. All the extracts inhibited the XO activities in a dose dependent manner. The IC_50_ concentrations of rapeseed meal, cottonseed meal and allopurinol (positive control) (Table 4) were significantly (*P* < 0.01) lower than that of the soybean meal. The results indicated that both rapeseed and cottonseed meal could be potential sources of bioactive compounds to inhibit the XO activity. Umamaheswari *et al.* [14] reported the contribution of phenolics and flavonoids toward XO inhibition through interaction in the reactive sites.

### 2.6. Tyrosinase Inhibitory Activity

**Figure 6:**
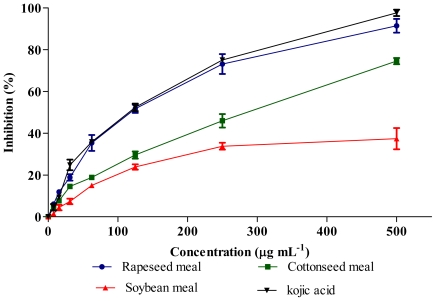
Tyrosinase inhibitory activities of meal extracts at different concentrations. Each value represents mean ± SEM of three replicates.

Tyrosinase enzyme activity was inhibited by meal extracts and kojic acid (positive control) in a dose dependent manner (Figure 6). The IC_50_ concentration of extracts (Table 4) showed significant difference (*P* < 0.01) and interestingly rapeseed meal extract showed the strongest inhibitory activity similar to kojic acid. The HPLC results indicated the presence of gallic acid in the rapeseed meal (Table 2) which has been implicated in the inhibition of tyrosinase activity. Kubo *et al.* [15] previously reported the strong tyrosinase inhibitory activity of gallic acid as compared to other phenolics and flavonoids. Furthermore, the antioxidant activity may also affect tyrosinase activity [16].

### 2.7. Anti-Inflammatory Activity

**Figure 7:**
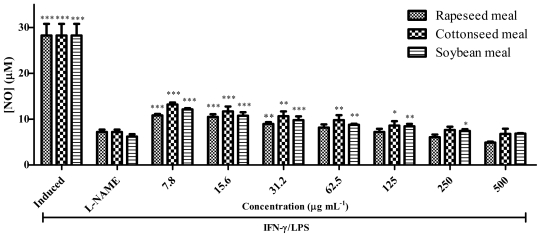
Effects of various concentrations of rapeseed meal, cottonseed meal and soybean meal extracts on NO production by IFN-γ/LPS stimulated RAW 264.7 cells. Each bar represents the mean ± standard error of three independent experiments. *** *P* < 0.0001; ** *P* < 0.001; * *P* < 0.01 indicates significant difference as compared to the control (L-NAME).

**Figure 8:**
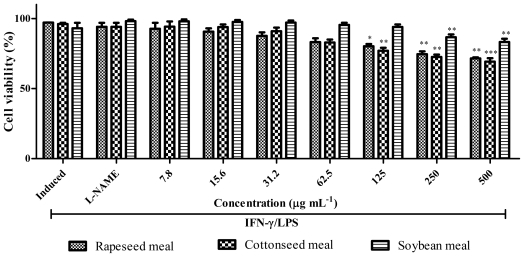
Effects of various concentrations of rapeseed meal, cottonseed meal and soybean meal extracts on cell viability of IFN-γ/LPS stimulated RAW 264.7 cells. Each bar represents the mean ± standard error of three independent experiments. *** *P* < 0.0001; ** *P* < 0.001; * *P* < 0.01 indicates significant difference as compared to the control (L-NAME).

The meal extracts were analysed for their inhibitory activity on nitric oxide (NO) production in RAW 264.7 cells induced by lipopolysaccharide (LPS) and interferon gamma (IFN-γ) as well as their effects on cell viability. Figures 7 and 8 show the NO inhibition and cell viability, respectively for rapeseed, cottonseed and soybean meals. Induced cells produced NO through inducible NO synthase (iNOS) as symptoms of inflammation. Rapeseed meal extract inhibited the NO production in a dose dependent manner and at 62.5 μg mL^−1^ could inhibit the NO production similar to *N*^ω^-nitro-l-arginine methyl ester (L-NAME). However the cell viability result (Figure 8) shows that cell viability significantly (*P* < 0.001) decreased at 125 to 500 μg mL^−1^. Therefore rapeseed meal extract at 62.5 μg mL^−1^ showed strong ability to inhibit the iNOS while maintaining cell viability comparable to L-NAME.

Cottonseed meal and soybean meal extracts also inhibited NO production equal to L-NAME but at a higher concentration as compared to that of rapeseed meal. Therefore, among meal extracts used in this study, rapeseed meal extract seemed to be promising as a source of anti-inflammatory compounds as it strongly inhibited iNOS, at the same time maintaining cell viability. The iNOS suppressing effect of the rapeseed could be due to the blocking of iNOS expression, inactivation of iNOS catalytic function or scavenging of NO radicals [17]. Various compounds could be involved in iNOS suppression by additive or synergistic effects although the roles of phenolics and flavonoids as main constituents of plant material responsible in iNOS suppression have been previously demonstrated [18,19].

The study conducted by Salvati *et al.* [20] indicated that rapeseed oil could prevent cognitive impairment of brain in Sprague-Dawley rats by improving the antioxidant status. They attributed this activity to the all isomeric structures of tocopherol such as α, β, δ, γ and coenzyme Q which are naturally present in the oil. Studies in human showed that α-tocopherol reduces oxidative stress and inflammation [21] and Chen *et al.* [22] also reported the synergistic effects of flavonoid and phenolic with α-tocopherol to enhance the antioxidant capacity than that provided by each compound separately. Therefore the antioxidant and anti-inflammatory activity of the rapeseed meal could be due to the presence of phenolics, and flavonoids, as well as different isomeric structures of tocopherol. However, the antioxidant and anti-inflammatory activities of various saturated and unsaturated fatty acids in the cottonseed and soybean oil have also been reported previously [23].

Table 5 shows the correlation analysis among parameters evaluated. It was observed that the phenolics and flavonoids contents were highly correlated to both antioxidant and anti-inflammatory activities with high correlation coefficient (*r*^2^) values ranging from 0.83 to 0.99. These results are consistent with those reported by Muanda *et al.* [24] and Lee *et al.* [25] who demonstrated the high correlations between phenolics and flavonoids, with antioxidant and anti-inflammatory activities. A linear correlation of free radical scavenging, NO scavenging and total antioxidant activities with the levels of phenolic and flavonoid compounds have also been reported previously [26].

Low positive correlations (*r*^2^ = 0.37–0.70) were observed between phenolics and flavonoids with xanthine and tyrosinase inhibitory activities in this study. Owan and Johns [27] and Wang *et al.* [28] also reported the low positive correlation of phenolics and flavonoids with xanthine and tyrosinase inhibitory activities.

## 3. Experimental Section

### 3.1. Samples and Microwave Extract Preparation

Rapeseed, cottonseed and soybean meals were purchased from Golden Jomalina Food Industries Sdn. Bhd. (Malaysia). The samples were ground into fine powder and 4 gram of each sample was extracted by using 20 mL of methanol and heated in a microwave (280 W) for 4 min. The extract obtained was filtered through filter paper (Whatman No. 1) and evaporated to dryness to obtain the crude extract for further analyses. The crude extracts were dissolved in dimethyl sulfoxide and the stock solutions were used for biological activity assays.

### 3.2. Total Phenolic and Flavonoid Compounds

#### 3.2.1. Chemicals

Methanol, hydrochloric acid, Folin-Ciocalteu reagent, sodium carbonate, aluminium chloride, sodium hydroxide, ascorbic acid, alpha-tocopherol, butylated hydroxytoluene (BHT), 1.1-diphenyl-2-picrylhydrazyl (DPPH), dimetyl sulfoxide (DMSO), and acetonitrile HPLC grade were purchased from Fisher Scientifics, USA. Dulbecco’s Modified Eagle Medium (DMEM), 3-4,5-dimethylthiazol-2,5-diphenyltetrazolium bromide thiazol blue (MTT), foetal bovine serum, phosphoric acid, sulfanilamide, naphtyl ethylene diaminedihydrochloride, *N*^ω^-nitro-l-arginine methyl ester (L-NAME), lipopolysaccharide (LPS), tyrosinase, xanthine oxidase and all phenolics and flavonoids standard were purchased from Sigma Aldrich and Interferon gamma (IFN-γ) was purchased from eBioscience, Inc. The other chemicals used in this study were bought from Merck.

#### 3.2.2. Total Phenolic Compounds

Total phenolic compounds was determined according to Halici *et al.* [29]. Briefly, 0.5 mL of each extract, 2.5 mL Folin-Ciocalteu reagent and 2 mL of 7.5% (w/v) Na_2_CO_3_ were mixed. The mixture was vortexed and incubated at room temperature for 90 min. The absorbance was read using a visible spectrophotometer (Novaspec II Visiblespectro) at 765 nm. The results were expressed as mg gallic acid equivalents g^−1^ dry weight (DW).

#### 3.2.3. Total Flavonoid Compounds

The total flavonoid compounds in each sample extract was determined according to Ismail *et al.* [30]. An aliquot (0.1 mL) of extract was added to 0.3 mL 5% (w/v) NaNO_2_ and incubated for 5 min. Then, 0.3 mL 10% (w/v) AlCl_3_ and 2 mL 1 M NaOH were added and the total volume was made up to 5 mL with distilled water. The absorbance was measured at 510 nm by using a visible spectrophotometer (Novaspec II Visiblespectro). The results were expressed as mg rutin equivalents g^−1^ DW.

#### 3.2.4. Analyses of Phenolic and Flavonoid Compounds by HPLC

The phenolic and flavonoid compounds of samples were quantitatively measured by high performance liquid chromatography (HPLC) as described by Crozier *et al.* [31], with some modification. Phenolic standards were gallic acid, syringic acid, vanillic acid, salicylic acid, and caffeic acid. Flavonoid standards were quercetin, rutin, myricetin, kaempferol, naringin and apigenin while isoflavonoid standards were genistein and daidzein. An aliquot of 20 μL from each extract was loaded on the HPLC (Agilent 1200) equipped with an analytical column Intersil ODS-3 (5μm 4.6 × 150 mm, Gl Science Inc.). Solvents comprising deionized water (solvent A) and acetonitrile (solvent B) were used. The pH of water was adjusted to 2.5 with trifluoroacetic acid. The phenolic and isoflavonoid compounds were detected at 280 nm while flavonoid compounds at 350 nm. The column was equilibrated by 85% solvent A and 15% solvent B. Then the ratio of solvent B was increased to 85% in 50 min followed by reducing the solvent B to 15% in 55 min and maintaining the ratio for another 5 min. The flow rate was 0.6 mL min^−1^.

### 3.3. Antioxidant Activity

#### 3.3.1. Free Radical Scavenging Activity

The free radical scavenging activity of the extract was determined using the DPPH assay as described by Gulcin *et al.* [32]. One milliliter methanolic extract of each meal at different concentrations was mixed with 3 mL 0.1 mM solution of 1,1-diphenyl-2-picrylhydrazil (DPPH) in methanol. After incubation at room temperature for 30 min in the dark, the absorbance of the mixture was read using a spectrophotometer (Novaspec II Visblespectro) at 517 nm. Ascorbic acid and α-tocopherol were used as antioxidant standards. Free radical scavenging activity from the sample was calculated according to the formula:

(1) $$[({\text{A}}_{0}-{\text{A}}_{1})/{\text{A}}_{0}]\times 100\%$$

where A_0_ was the absorbance of the control reaction and A_1_ was the absorbance in the presence of the sample.

#### 3.3.2. Nitric Oxide Scavenging Activity

The nitric oxide (NO) scavenging activity of each plant extract was determined by the method of Tsai *et al.* [17]. Sixty microliters of two-fold diluted sample were mixed with 60 μL of 10 mM sodium nitroprusside in phosphate buffered saline (PBS) in a 96-well flat-bottomed plate and incubated under light at room temperature for 150 min. Finally, an equal volume of Griess reagent was added into each well in order to measure the NO content. Ascorbic acid and α-tocopherol were used as controls. The NO scavenging activity was calculated according to the formula: [(A_0_ − A_1_)/A_0_] × 100%; where A_0_ was the absorbance of the control reaction and A_1_ was the absorbance in the presence of the sample.

#### 3.3.3. Total Antioxidant Activity Assay

*Ferric Thiocyanate (FTC) Test.* This test was carried out according to the method described by Ismail *et al.* [30]. The absorbance of the samples was read at 500 nm by using a spectrophotometer (Molecular Devices Inc., USA). This procedure was repeated every 24 h until the control sample reached its maximum absorbance value. Butylated hydroxytoluene (BHT) was used as standard antioxidant in this test.

*hiobarbituric Acid (TBA) Test.* This test was carried out according to Ismail *et al.* [30], once the control sample from FTC test reached its maximum absorbance value. The absorbance of the reaction was measured at 532 nm using a spectrophotometer (Molecular Devices Inc., USA).

### 3.4. Xanthine Oxidase Inhibitory Activity

The xanthine oxidase (XO) inhibitory activity was performed based on Orhan *et al.* [33]. Twenty microliters XO (0.003 unit/well) dissolved in phosphate buffer (0.1 M, pH = 7.5) were mixed with various concentrations of each sample in 10 μL of DMSO in a 96-well plate and incubated for 10 min at room temperature. Twenty microliters of 0.1 mM xanthine was added to the mixture. The uric acid formation was measured by a spectrophotometer (Molecular Devices Inc., USA) at 295 nm. Allopurinol was used as a positive control.

### 3.5. Tyrosinase Inhibitory Activity

The tyrosinase inhibitory activity of the extracts were determined based on Lee *et al.* [34]. Briefly, the extracts were serially diluted with phosphate buffer (50 mM) in 96-well microtiter plate. Equal volume of tyrosinase (SIGMA) (333 units mL^−1^) was added into wells. After 5 min incubation at room temperature, L-DOPA (6 mM) was added. The absorbance was measured at 492 nm using a spectrophotometer (Molecular Devices Inc., USA).

### 3.6. Anti-Inflammatory Activity

The murine monocytic macrophage RAW 264.7 cell line (European Cell Culture Collection, CAMR, UK) was cultured in Dulbecco’s Modified Eagle Media (DMEM) (2 mM l-glutamine, 45 g L^−1^ glucose, 1 mM sodium pyruvate) with 10% fetal bovine serum (FBS). The cells were cultured at 37 °C with 5% CO_2_ and were subcultured twice a week. The cells were seeded in 96-well tissue culture plates (1 × 10^6^ cells mL^−1^) and incubated for 24 h at 37 °C with 5% CO_2_. Then, 100 μL of test extract in DMSO was then added and serially diluted to give a final concentration of 200 μg mL^−1^ in 0.1 % DMSO. Cells were then stimulated with 200 U mL^−1^ of recombinant mouse interferon-gamma (IFN-γ) and 10 μg mL^−1^ *Escherichia coli* lipopolysaccharide (LPS) and incubated at 37 °C for another 17 h. The presence of NO was determined in cell culture medium by Griess reagent and cell viability was detected by using MTT cytotoxicity assay as described by Ahmad *et al.* [35]. *N*^ω^-nitro-l-arginine methyl ester (L-NAME) was used as iNOS inhibitor (control) at a concentration of 250 μM.

### 3.7. Statistical Analysis

Statistical analysis was conducted using GLM procedure [36] using a complete randomized design following the model: Yi = μ + Ti + ei, where μ is the mean value, Ti is the treatment effect and ei is the experimental error, respectively. Differences in LSD were considered significant at *P* < 0.05. GraphPad Prism 5 software [37] was used for all the statistical analyses in anti-inflammatory assay. The correlation coefficients among parameters studied were obtained by using the MS Excel software.

## 4. Conclusions

The results obtained in this study showed that rapeseed meal as an agro-industrial by-product exhibited notable antioxidant, anti-inflammatory, xanthine and tyrosinase inhibitory activities when compared to cottonseed and soybean meals. Correlation analysis showed that phenolics and flavonoids contents were highly correlated to both antioxidant and anti-inflammatory activities with high correlation coefficient (*r*^2^) values ranging from 0.83 to 0.99. Therefore rapeseed meal was found to be promising as a natural source of bioactive compounds suitable for the food and feed industries and for the development of various pharmaceutical and value-added products.

## Figures and Tables

**Table 1:** Total phenolic and flavonoid compounds.

Samples	Total Phenolic Compounds *	Total Flavonoid Compounds **
Rapeseed meal	5.3 ^a^	2.3 ^a^
Cottonseed meal	1.5 ^b^	1.2 ^b^
Soybean meal	0.9 ^c^	0.8 ^c^

SEM	0.09	0.07

*P* value	0.01	0.01

* mg gallic acid equivalents g^−1^ DW;

** mg rutin equivalents g^−1^ DW; Each value represents mean of three replicates; SEM: standard error of the mean; Means in the same column with different superscripts are significantly different (*p* < 0.01).

**Table 2:** Phenolic and flavonoid compounds determined by HPLC a.

Compounds	Phenolic, Flavonoid and Isoflavonoid (μg g^−1^ DW)		
	Rapeseed Meal	Cottonseed Meal	Soybean Meal
**Phenolics**			

Gallic acid	419.5 ± 3.32	nd	nd
Salicylic acid	nd	nd	nd
Caffeic acid	nd	nd	295.8 ± 2.73
Vanillic acid	nd	nd	nd
Syringic acid	177.6 ± 2.82	nd	nd

**Flavonoids**			

Apigenin	64.9 ± 2.48	nd	nd
Kaempferol	494.0 ± 1.71	113.7 ± 4.59	nd
Myricetin	nd	nd	nd
Naringenin	793.5 ± 5.90	178.9 ± 6.88	352.3 ± 3.66
Quercetin	nd	nd	nd
Rutin	nd	209.1 ± 2.45	nd

**Isoflavonoids**			

Daidzein	nd	nd	521.4 ± 6.58
Genistein	nd	nd	nd

a High performance liquid chromatography; nd: Not detected; Each value represents mean ± SEM of three replicates.

**Table 3:** The IC_50_ values of extracts and vitamins in DPPH and nitric oxide scavenging activities.

Samples	IC_50_ (μg mL^−1^)	
	DPPH Scavenging Activity	Nitric Oxide Scavenging Activity
Rapeseed meal	87.9 ^c^	108.2 ^b^
Cottonseed meal	191.1 ^b^	191.3 ^a^
Soybean meal	466.3 ^a^	207.6 ^a^
Vitamin C	20.4 ^d^	52.7 ^c^
Vitamin E	45.4 ^d^	72.9 ^c^

SEM	7.98	7.12

*P* value	*P* < 0.01	*P* < 0.01

Means in the same column with the different superscripts are significantly different at *P* < 0.01; Analyses were done in triplicate; SEM: standard error of the mean.

**Table 4:** The IC_50_ values of xanthine oxidase and tyrosinase inhibitory activities of meal extracts and positive control.

Samples	IC_50_ μg mL^−1^	
	Xanthine Oxidase Inhibitory Activity	Tyrosinase Inhibitory Activity
Rapeseed meal	84.8 ^b^	120.3 ^c^
Cottonseed meal	86.8 ^b^	286.0 ^b^
Soybean meal	464.2 ^a^	>500 ^a^
Allopurinol	29.7 ^c^	-
Kojic acid	-	116.2 ^c^

SEM	7.55	5.43

*P* value	*P* < 0.01	*P* < 0.01

Means in the same column with different superscripts are significantly different at *P* < 0.01.

**Table 5:** Relationship between TPC, TFC, DPPH, NO, XO, TI, FTC, TBA and iNOS of meal extracts.

Correlation (*r*^2^)									
Parameters	TPC	TFC	DPPH	NO	FTC	TBA	XO	TI	iNOS
TPC	-	0.987	0.637	0.998	0.877	0.839	0.373	0.702	0.854
TFC	-	-	0.734	0.993	0.933	0.911	0.482	0.596	0.922
DPPH	-	-	-	0.656	0.911	0.939	0.933	0.111	0.932
NO	-	-	-	-	0.898	0.863	0.397	0.677	0.873
FTC	-	-	-	-	-	0.997	0.714	0.359	0.998
TBA	-	-	-	-	-	-	0.762	0.307	0.999
XO	-	-	-	-	-	-	-	0.006	0.749
TI	-	-	-	-	-	-	-	-	0.321
iNOS	-	-	-	-	-	-	-	-	-

TPC: total phenolic compounds; TFC: total flavonoid compounds; DPPH: DPPH scavenging test; NO: Nitric oxide scavenging test; XO: xanthine oxidase inhibitory test; TI: tyrosinase inhibitory test; FTC: ferric thiocyanate test; TBA: thiobarbituric acid test; iNOS: induced nitric oxide synthase.
